# *CYP2C19* Polymorphisms in Indonesia: Comparison among Ethnicities and the Association with Clinical Outcomes

**DOI:** 10.3390/biology10040300

**Published:** 2021-04-06

**Authors:** Muhammad Miftahussurur, Dalla Doohan, Ari Fahrial Syam, Iswan Abbas Nusi, Phawinee Subsomwong, Langgeng Agung Waskito, Hasan Maulahela, Fardah Akil, Willy Brodus Uwan, Gontar Siregar, Kartika Afrida Fauzia, Yudith Annisa Ayu Rezkitha, Abdul Rahman, I Dewa Nyoman Wibawa, Alexander Michael Joseph Saudale, Marselino Richardo, Titong Sugihartono, Alvi Chomariyati, Taufan Bramantoro, Tomohisa Uchida, Yoshio Yamaoka

**Affiliations:** 1 Gastroentero-Hepatology Division, Department of Internal Medicine, Faculty of Medicine-Dr. Soetomo Teaching Hospital, Universitas Airlangga, Surabaya 60286, Indonesia; iswan-a-n@fk.unair.ac.id (I.A.N.); titong.sugihartono@fk.unair.ac.id (T.S.); alvi.chomariyati-2017@fk.unair.ac.id (A.C.); 2 Institute of Tropical Disease, Universitas Airlangga, Surabaya 60115, Indonesia; doctordoohan@gmail.com (D.D.); langgengaw@gmail.com (L.A.W.); kartikafauzia@gmail.com (K.A.F.); yudithannisaayu@gmail.com (Y.A.A.R.); 3 Department of Environmental and Preventive Medicine, Oita University Faculty of Medicine, Yufu 879-5593, Japan; phawinee@oita-u.ac.jp; 4 Division of Gastroenterology, Department of Internal Medicine, Faculty of Medicine, University of Indonesia, Jakarta 10430, Indonesia; ari_syam@hotmail.com (A.F.S.); hasan.maulahela@yahoo.com (H.M.); 5 Department of Microbiology and Immunology, Hirosaki University Graduate School of Medicine, Hirosaki, Aomori 036-8562, Japan; 6 Center of Gastroentero-Hepatology, Department of Internal Medicine, Faculty of Medicine, Hasanuddin University, Makassar 90245, Indonesia; dndakil@gmail.com; 7 Department of Internal Medicine, Santo Antonius Hospital, Pontianak 78243, Indonesia; uwan.willyb@gmail.com; 8 Division of Gastroentero-Hepatology, Department of Internal Medicine, Faculty of Medicine, University of Sumatra Utara, Medan 20155, Indonesia; gontarsiregar@gmail.com; 9 Faculty of Medicine, University of Muhammadiyah Surabaya, Surabaya 60113, Indonesia; 10 Department of Internal Medicine, Kolaka General Hospital, Kolaka 93511, Indonesia; arahman22272@gmail.com; 11 Division of Gastroentero-Hepatology, Department of Internal Medicine, Faculty of Medicine, University of Udayana, Denpasar 80232, Indonesia; agusbobwibawa@yahoo.com; 12 Department of Internal Medicine, Prof. Dr. W. Z. Johannes General Hospital, Kupang 85111, Indonesia; alexsaudale10@gmail.com; 13 Department of Internal Medicine, Merauke City General Hospital, Merauke 99613, Indonesia; marselino_richardo@yahoo.com; 14 Department of Dental Public Health, Faculty of Dental Medicine, Universitas Airlangga, Surabaya 60131, Indonesia; taufan-b@fkg.unair.ac.id; 15 Department of Molecular Pathology, Oita University Faculty of Medicine, Yufu 879-5593, Japan; tomohisa@oita-u.ac.jp; 16 Global Oita Medical Advanced Research Center for Health, Oita University, Yufu 879-5593, Japan; 17 Department of Gastroenterology and Hepatology, Baylor College of Medicine, Houston, TX 77030, USA

**Keywords:** *H. pylori*, gastritis, *CYP2C19*, polymorphism, infectious disease

## Abstract

**Simple Summary:**

CYP2C19 is known as an enzyme primarily responsible for metabolizing various drugs, such as proton pump inhibitor, antiplatelet, anti-epileptic, and anticoagulant. *CYP2C19* is known to be polymorphic and can result in the clinical efficacy of drugs. To examine the prevalence and the distribution of the *CYP2C19* genetic polymorphisms in Indonesia, we performed polymerase chain reaction-restriction fragment length polymorphism to the genomic DNA of Indonesian participants. In addition, we also analyzed the distribution of *CYP2C19* polymorphisms among ethnicities and clinical outcomes. We found that the prevalence of intermediate metabolizers were the highest in Indonesia, followed by rapid metabolizers and poor metabolizers, respectively. The distribution of metabolizer groups were different between ethnic groups in Indonesia. Therefore, dosage adjustment should be considered when administering drugs-affected by CYP2C19 in Indonesia. The results presented in this study showed the distribution of *CYP2C19* variant alleles at the population level in Indonesia and might be used as a consideration for providing personalized treatment in clinical practice.

**Abstract:**

*CYP2C19* polymorphisms are important factors for proton pump inhibitor-based therapy. We examined the *CYP2C19* genotypes and analyzed the distribution among ethnicities and clinical outcomes in Indonesia. We employed the polymerase chain reaction-restriction fragment length polymorphism method to determine the *CYP2C19* genotypes and evaluated inflammation severity with the updated Sydney system. For CYP2C19*2, 46.4% were the homozygous wild-type allele, 14.5% were the homozygous mutated allele, and 39.2% were the heterozygous allele. For CYP2C19*3, 88.6% were the homozygous wild-type allele, 2.4% were the homozygous mutated allele, and 9.0% were the heterozygous allele. Overall, the prevalence of rapid, intermediate, and poor metabolizers in Indonesia was 38.5, 41.6, and 19.9%, respectively. In the poor metabolizer group, the frequency of allele *2 (78.8%) was higher than the frequency of allele *3 (21.2%). The Papuan had a significantly higher likelihood of possessing poor metabolizers than the Balinese (OR 11.0; P = 0.002). The prevalence of poor metabolizers was lower compared with the rapid and intermediate metabolizers among patients with gastritis and gastroesophageal reflux disease. Intermediate metabolizers had the highest prevalence, followed by rapid metabolizers and poor metabolizers. Dosage adjustment should therefore be considered when administering proton pump inhibitor-based therapy in Indonesia.

## 1. Introduction

Triple therapy consisting of two antibiotics, such as amoxicillin and clarithromycin, accompanied by a proton pump inhibitor (PPI) is widely employed for the eradication therapy of *Helicobacter pylori* infection [1,2,3,4], an eradication that can reduce gastric mucosal inflammation, and show improvement in chronic gastritis [4,5,6,7]. PPIs increase *H. pylori* sensitivity to the effects of antibiotics by raising the intragastric pH to near neutral levels [8] and by increasing the intragastric concentration of antibiotics by decreasing antibiotic decay in digestive fluids [8,9]. There is consensus that PPI is an important drug for first-, second-, and even third-line therapy for *H. pylori* eradication [1,4].

PPI is an acid-activated prodrug that is inactive in its native form [10] and is mainly metabolized in the liver. CYP2C19 is an enzyme primarily responsible for metabolizing most PPIs, including omeprazole, esomeprazole, lansoprazole, and pantoprazole [8], with the exception of rabeprazole, which is primarily metabolized by non-enzymatic reduction to form thioether [9]. CYP2C19 plays an important role in converting these PPIs into hydroxyl compounds through aromatic hydroxylation reactions before finally being metabolized by CYP3A4 into sulfone [8,9,11].

The *CYP2C19* gene contains nine exons and is located in chromosome 10 (10q24.1-10q24.3) [8]. The protein is expressed mainly in the liver and, to a lesser degree, in the intestinal wall. *CYP2C19* is known to be polymorphic and presents 19 variants [11]. *CYP2C19* most frequently shows two types of mutated alleles: CYP2C19*2 and CYP2C19*3. The most common mutation of CYP2C19*2 contains a single base G to A mutation at position 681 in exon 5, a mutation that produces splicing and changes the open reading frame, generating an early stop codon and a truncated protein. CYP2C19*3 contains a single base G to A mutation at position 636 in exon 4, which also consequently generates an early stop codon and a truncated protein [9,12].

Genetic polymorphisms of *CYP2C19* can result in differing pharmacokinetics, pharmacodynamics, and consequently differences in the clinical efficacy of PPIs [13,14,15]. According to their *CYP2C19* genotype differences, individuals can be characterized into three metabolic types: rapid, intermediate, and poor metabolizers. Rapid metabolizers are characterized by having two wild-type alleles and rapid enzyme activity, whereas poor metabolizers are characterized by two mutant alleles and an extremely slow rate of enzyme activity. The intermediate metabolizer is characterized by one wild-type and one mutant allele and, therefore, tends to have a moderate rate of enzyme activity. Although forming mostly a minority group, the poor metabolizer phenotype shows wide inter-ethnic differences between populations (13–20% for the Japanese [16,17,18], 10.4% for Thais [19], 11–15% for the Chinese [14,20], 2–5% for Europeans [21,22,23], and 9% for Egyptians [24]). This phenotype might therefore have wide differences in terms of drug metabolism among ethnicities. Several studies have been conducted on the effect of the *CYP2C19* genotype on curing *H. pylori* infection in various populations. This defect directly affects enzyme activity and consequently influences the efficacy of therapy. Due to its importance, dosing guidelines for drugs such as clopidogrel, tricyclic antidepressants, and selective serotonin-reuptake inhibitors have been established based on *CYP2C19* genotypes [25,26,27,28]. Identifying the genetic variation of *CYP2C19* is an important factor in the dose adjustment for PPIs and might therefore improve the eradication rate.

Indonesia is an archipelago country in Southeast Asia, between the Indian and Pacific oceans, with more than 13,000 islands populated by over 261 million people consisting of hundreds of native ethnic groups. A study in Indonesia reported *CYP2C19* polymorphism but evaluated CYP2C19*17 and only included one ethnicity (Bugis) as the participants [29]. Therefore, the CYP2C19*2 and CYP2C19*3 polymorphism variants present in the other ethnicities in Indonesia remain undiscovered. In addition, dyspepsia was the sixth and fifth most prevalent disease in Indonesian outpatients and inpatients, respectively [30]. Genotyping *CYP2C19* polymorphisms could be an important consideration when deciding on PPI therapeutic doses and for reducing dyspeptic symptoms. In this study, we examined the prevalence of the CYP2C19*2 and CYP2C19*3 genetic polymorphisms in Indonesia. We also analyzed the distribution among ethnicities and clinical outcomes.

## 2. Materials and Methods

### 2.1. Study Participants

**Figure 1:**
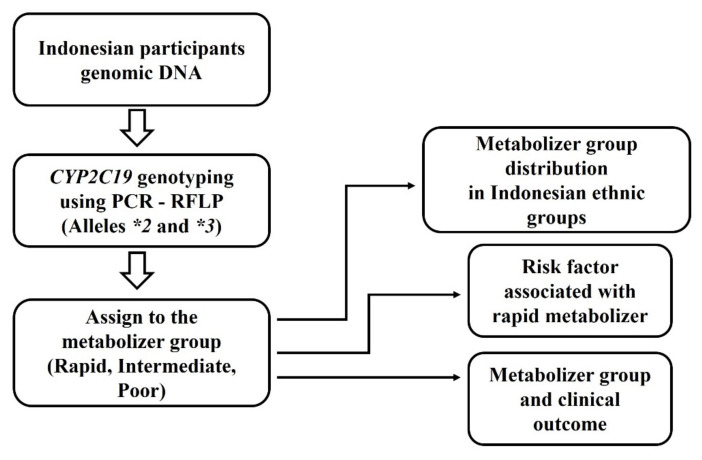
The systematic flow of methods and analyses in this study.

We used 784 samples from our previous nationwide studies in Indonesia conducted from January 2014 to September 2016 [31], as well as the diagnosis of various gastroduodenal diseases and gastric mucosal histological data. We randomly selected 166 participants based on ethnic groups and location, regardless of the result of the *H. pylori* infection status. The sample included Javanese (28 samples), Batak (27 samples), Bugis (37 samples), Chinese Indonesian (17 samples), Balinese (25 samples), Dayak (10 samples), Papuan (14 samples), and Timorese (8 samples). Upper endoscopies were performed and biopsies were taken for the histology and DNA analysis. Figure 1 shows the systematic flow of methods and analyses in this study. The study protocol was approved by the Ethics Committee of Dr. Soetomo Teaching Hospital (Surabaya, Indonesia), Dr. Cipto Mangunkusumo Teaching Hospital (Jakarta, Indonesia), Dr. Wahidin Sudirohusodo Teaching Hospital (Makassar, Indonesia), and Oita University Faculty of Medicine (Yufu, Japan). The study’s protocol followed the principles of the 2013 Declaration of Helsinki.

### 2.2. Endoscopy Diagnosis and Gastritis Severity Score

Clinical outcomes were obtained from the endoscopy diagnosis and gastritis severity scores by histology examination. The endoscopy diagnosis was reached based on the observations of the attending gastroenterologist and classified into gastritis, gastric ulcer, duodenal ulcer, and gastroesophageal reflux disease.

Gastric biopsy specimens were prepared and examined by the same pathologist (TU). Briefly, the paraffin-embedded biopsy was sliced and stained with hematoxylin-eosin and May–Giemsa staining. The inflammation severity was evaluated with a score of 0 (normal), 1 (mild), 2 (moderate), or 3 (marked) according to the updated Sydney system [32]. Antrum-predominant or corpus-predominant gastritis was determined if the total of the acute (neutrophil) and chronic (monocyte) inflammatory scores was higher in the antrum or corpus, respectively [33].

### 2.3. CYP2C19 Polymorphism Genotyping

For the genomic DNA isolation, we employed 150 µL of gastric homogenate extracted using the DNeasy Blood and Tissue Kit (Qiagen, Germany) according to the manufacturer’s instructions. We evaluated the CYP2C19*2 (rs4244285; 681G>A and CYP2C19*3 (rs4986893; 636G>A) by polymerase chain reaction (PCR)-restriction fragment length polymorphism. We employed genomic DNA as a template with specific primers and conditions, as previously described [16,34]. Amplification of CYP2C19*2 resulted in a 168 bp band, and amplification of CYP2C19*3 resulted in a 119 bp band. The amplified PCR products of CYP2C19*2 and *3 were digested with *Sma*I restriction enzyme (New England Biolabs, Tokyo, Japan) for 1 h at 25 °C and *Bam*HI restriction enzyme (Takara, Tokyo, Japan) for 1 h at 30 °C, respectively. The digested product was checked by agarose gel electrophoresis stained with ethidium bromide. Given that the restriction site is absent in the mutated alleles, the PCR products are not digested by the enzyme.

### 2.4. Classification of CYP2C19 Genotype Groups

Based on the CYP2C19*2 genotyping results, we categorized the patients into three different genotypes: wild-type allele (*1/*1), homozygous for the mutated allele (*2/*2), and heterozygous for the mutated allele (*1/*2), and for the CYP2C19*3 polymorphism, we categorized the patients into three genotypes: wild-type allele (*1/*1), homozygous for the mutated allele (*3/*3), and heterozygous for the mutated allele (*1/*3). Based on the combination of *CYP2C19* polymorphism genotyping in exons 5 and 4, the enzymatic activity associated with *CYP2C19* genotypes was classified into three groups: rapid metabolizer, intermediate metabolizer, and poor metabolizer. Homozygous wild-type alleles (*1/*1) in both exons were regarded as rapid metabolizers. Heterozygous allele in one exon (*1/*2 or *1/*3) while the other exon being wild-type allele was regarded as intermediate metabolizers. Homozygous mutant allele in one exon (*2/*2 or *3/*3) while the other exon being wild-type allele was regarded as poor metabolizers. Individual with a mutation in both exon 5 and exon 4 (*2/*2 and *1/*3 genotypes, *1/*2 and *3/*3 genotypes, or *1/*2 and *1/*3 genotypes) was assigned as heterozygous (*2/*3) and also regarded as a poor metabolizers.

### 2.5. Statistical Analysis

The statistical analysis was performed using SPSS version 23 (IBM Corp., Armonk, NY, USA). The significance of the difference in *CYP2C19* genotypes between ethnicities was analyzed using the chi-squared test or Fisher’s exact test. The odds ratios (ORs) with 95% confidence intervals (CIs) were calculated using a logistic regression model. A P-value < 0.05 was regarded as statistically significant. The observed genotype frequencies were compared with expected values calculated with the Hardy–Weinberg Equilibrium equation (*p*^2^ + 2*pq* + *q*^2^ = 1) by using a chi-squared test with a degree of freedom of 1, in which *p* represents the frequency of the wild-type allele and *q* represents the frequency of the mutated (variant) allele. The allele frequencies of multiple genes was also calculated using Hardy–Weinberg Equilibrium equation (*p*^2^ + *q*^2^ + *r*^2^ + 2*pq* + 2*pr* + 2*qr*) = 1, in which *p* represents the frequency of the wild-type allele and *q* and *r* represent the frequency of the mutated allele.

## 3. Results

### 3.1. Demographic Data and Endoscopy Results

This study enrolled 166 patients (101 men and 65 women; mean age, 46.7 ± 14.2 years; range, 17–80 years). Table 1 presents the demographic data. Based on the ethnic groups, there were 14 Papuan, 27 Batak, 25 Balinese, 10 Dayak, 28 Javanese, 37 Bugis, 17 Chinese, and 8 Timorese participants. Among them, 143 (143 out of 166; 86.1%) patients were endoscopically diagnosed with gastritis, 5 (5 out of 166; 3.0%) patients with gastric ulcers, 1 (1 out of 166; 0.6%) patient with duodenal ulcer, and 17 (17 out of 166; 10.2%) with gastroesophageal reflux disease (GERD).

### 3.2. CYP2C19 Polymorphism Genotyping

Table 2 shows the genotyping results. For CYP2C19*2, we observed that 77 (46.4%) of the 166 patients had the homozygous wild-type allele (*1/*1); in total, 24 (14.5%) had the homozygous mutated allele (*2/*2); a total of 65 (39.2%) had the heterozygous allele (*1/*2). As for the CYP2C19*3 genotyping results, we observed that 147 (88.6%) patients had the homozygous wild-type allele (*1/*1); in total, 4 (2.4%) had the homozygous mutated allele (*3/*3); a total of 15 (9.0%) had the heterozygous allele (*1/*3).

In this population genetic study, we employed the Hardy–Weinberg equation to determine whether the observed genotype frequencies in the Indonesian population differed from the frequencies predicted by the equation. The *p*-value for CYP2C19*2 (representing *1 allele) was 0.66, and the *q*-value (representing *2 allele) was 0.34. As for CYP2C19*3, the *p*-value (representing *1 allele) was 0.93, and the *q*-value (representing *3 allele) was 0.07. We found that the CYP2C19*2 genetic variant conformed to the equation (*X*^2^ = 2.72, P = 0.099), whereas the CYP2C19*3 genetic variant showed a pattern that did not fit the equation (*X*^2^ = 14.87, P < 0.001), suggesting that the genotype frequency of CYP2C19*3 was not in equilibrium.

We expected that the *CYP2C19* phenotype would be determined based on the combination of CYP2C19*2 and CYP2C19*3 polymorphism genotyping; we found six different allelic combinations (Table 2). In total, 64 (38.6%) of the 166 patients were homozygous for the wild-type allele in both exon 5 and exon 4 (*1/*1); overall, 36.1% (60 out of 166) were heterozygous in exon 5 (*1/*2); a total of 9 (5.4%) were heterozygous in exon 4 (*1/*3). Overall, 6 (3.6%) of the 166 patients were heterozygous in exons 5 and 4 (*2/*3); a further 23 (13.9%) were homozygous for the mutated allele in exon 5 (*2/*2); a total of 4 (2.4%) were homozygous for the mutated allele in exon 4 (*3/*3). We calculated that for all *CYP2C19* genotypes, 59.3% (197 out of 332) were allele *1; in total, 33.7% (112 out of 332) were allele *2; in total, 6.9% (23 out of 332) were allele *3. We also employed Hardy–Weinberg equation to analyze the expected frequencies of all *CYP2C19* genotypes. We found that the *p*-value (representing *1 allele) was 0.5934, the *q*-value (representing *2 allele) was 0.3373, and the *r*-value (representing *3 allele) was 0.0693; suggesting that in general, *CYP2C19* did not fit to the equation (*X*^2^ = 16.91, P < 0.001).

The frequency of the recessive gene responsible for the decreased activity of CYP2C19 (alleles *2 and *3) was 40.7% (135 out of 332). Overall, the prevalence of rapid, intermediate, and poor metabolizers in Indonesia was 38.5, 41.6, and 19.9%, respectively, suggesting that more than half (61.5%) of the participants had reduced *CYP2C19* enzyme capability. In the poor metabolizer group, the frequency of allele *2 (52 out of 66; 78.8%) was higher than the frequency of allele *3 (14 out of 66; 21.2%), suggesting that poor metabolizers of CYP2C19 in Indonesia possess a mutation predominant in exon 5 (Table 2).

### 3.3. CYP2C19 Polymorphisms between Indonesian Ethnicities

Table 3 shows the *CYP2C19* genotype comparison between eight ethnic groups in Indonesia. Each ethnic group had a different distribution of *CYP2C19* genotyping. Based on ethnicity, the Balinese participants had the highest prevalence for rapid metabolizers (52.0%), followed by Javanese (46.4%), Dayak (40%), Bugis (37.8%), Timorese (37.5%), Batak (37.0%), Chinese (29.4%), and Papuan (14.3%) participants, the ethnicity with the lowest prevalence of rapid metabolizers. Conversely, the Papuan participants had the highest prevalence of poor metabolizers (57.1%), whereas there were no poor metabolizers in the Timorese participants. The Balinese participants had a statistically significant higher likelihood of rapid metabolizers than the Papuan (Balinese: P = 0.030, OR 76.5, 95% CI 1.199–35.230) (Table 4). We also found that the Papuan participants had a significantly higher likelihood of poor metabolizers than the Balinese (P = 0.002, OR 11.0, 95% CI 2.498–48.433) (Supplementary Table S1).

### 3.4. CYP2C19 Polymorphisms and Clinical Outcomes

We classified the patients into four groups of clinical outcomes based on endoscopic observation: gastritis, gastric ulcer, duodenal ulcer, and GERD. Table 5 shows the distribution of enzyme metabolizer groups based on the clinical outcomes. Among the patients with gastritis, we found that the prevalence of poor metabolizers was lower than that of rapid and intermediate metabolizers. The prevalence of poor metabolizers was also lower than that of rapid and intermediate metabolizers in GERD, although this difference was not statistically significant. There were five participants with gastric ulcers; however, none of them were included in the poor metabolizer group. There was only one participant with duodenal ulcer, and they belonged to the poor metabolizer group.

We also analyzed the association between the *CYP2C19* metabolizer group and the histological scores based on the updated Sydney system (Supplementary Table S2). There was no significant association between neutrophil and monocyte infiltration and the metabolizer group (P > 0.05). There was no significant association between antrum-predominant and corpus-predominant gastritis and the metabolizer group (P > 0.05).

## 4. Discussion

*CYP2C19* polymorphism is widely reported to be associated with the efficacy of acid-related disease in the stomach due to the importance of S-mephenytoin-hydroxylase in the metabolism of various PPI drugs [8,35,36]. We found that, in Indonesia, intermediate metabolizers had the highest prevalence, followed by rapid metabolizers and poor metabolizers. In general, the prevalence of rapid metabolizers and poor metabolizers was 38.5 and 19.9%, respectively. Our results agree with previous studies that reported a prevalence of rapid metabolizers of 65–78% for whites but only 30–40% for Asian populations, whereas the prevalence of poor metabolizers in Asian populations (13–23%) was higher than in whites (2–5%) [16,20,21,36,37]. Although studies on *CYP2C19* polymorphisms in Indonesia are still scarce, the expected genotype distribution in this study showed the same pattern as another neighboring country in Southeast Asia (Thailand) and as other Asian countries, such as Korea, Japan, and China (Supplementary Table S3).

Although the prevalence of rapid, intermediate, and poor metabolizers in Indonesia was in line with that of the neighboring country and of Asians in general, a different pattern was also observed among ethnicities. These results also support the fact that ethnicity and geography play roles in *CYP2C19* polymorphism. Papuans, an ethnicity native to Papua Island, had a considerably higher prevalence of poor metabolizers than the other ethnicities, a result consistent with previous studies conducted in Papua New Guinea, a neighboring country that occupies the eastern half of the island of Papua, which reported a high prevalence of poor metabolizers [38,39]. The Papuan participants had the highest prevalence of poor metabolizers, whereas the Balinese participants had a significantly higher likelihood of having the rapid metabolizing enzyme than the Papuan. Dosage adjustment should therefore be considered for these ethnicities when administering PPI-based therapy. For rapid metabolizers, PPI-based therapy might have lower efficacy due to the faster metabolism of PPI; therefore, some patients might not respond to PPI-based therapy [40,41]. Increasing the PPI dosage in a population with a high prevalence of *CYP2C19* rapid metabolizers might provide a benefit in maintaining adequate concentrations and effectivity of the PPI. A study in Japan showed higher eradication rates for personalized *H. pylori* eradication therapy among different phenotypes (rapid metabolizer, 30 mg t.i.d.; poor metabolizer, 15 mg b.i.d.; intermediate metabolizer, 15 mg t.i.d.) compared with standard *H. pylori* eradication therapy (lansoprazole 30 mg b.i.d.) [35]. In contrast, the same dose of pantoprazole 40 mg in different phenotypes in South Korea had reduced efficacy in eradicating *H. pylori* in rapid metabolizers [36].

The prevalence of gastroduodenal disease was higher in the rapid and intermediate metabolizers than in the poor metabolizers, although the difference was not statistically significant. Our observation also showed an association between chronic pangastritis and intermediate metabolizers. In addition, the Papuan participants had the lowest prevalence of gastritis based on monocyte infiltration. These data support the hypothesis of the metabolizing enzyme affecting the prevalence of gastritis. Consistent with the results of a study in Thailand [16,42], more symptoms and diseases might develop due to the ineffectiveness of PPI therapy. Conversely, poor metabolizers had a slower rate of PPI metabolism, thereby increasing the peak-plasma concentration [43,44] and consequently increasing the mucosal gastroprotection from nonsteroidal anti-inflammatory drugs and other drugs, leading to a lower risk of developing gastritis, peptic ulcer disease, and peptic ulcer bleeding [19].

In this study, the CYP2C19*2 polymorphism conformed to the Hardy–Weinberg Equilibrium, which can demonstrate the validity of the genetic association study because, theoretically, the population should fit within the Hardy–Weinberg Equilibrium [45]. However, the application of the Hardy–Weinberg Equilibrium as the only tool for controlling the validity of population genetic studies has been controversial [46,47]. In this study, CYP2C19*3 did not conform to the equilibrium because of the high prevalence of the wild type, a deviation that might indicate a problem due to nonrandom mating, population stratification, admixture, and selection bias, although these could not be confirmed in the current study [45,48]. Another possible reason for the population not fitting within the Hardy–Weinberg Equilibrium is the small number of samples examined, which in this case was also one of the study’s limitations. Caution should therefore be taken when interpreting the results. In addition, the prevalence of rapid and poor metabolizer groups in Indonesia having a similar pattern to the *CYP2C19* genotyping results in other Asian countries, such as Japan and South Korea [16,42], showed that the geographical factor might also be related to the pattern.

There are several limitations in our study. First, the small number of participants and diseases, such as gastric ulcer and duodenal ulcer. Our results might therefore be insufficient for explaining the association between the disease and *CYP2C19* polymorphism. Further study is needed on *CYP2C19* polymorphisms with sufficiently large sample sizes in each ethnicity and disease. Second, this study primarily described the distribution of *CYP2C19* genotypes in the Indonesian population, and we did not examine the effect of the phenotypes on the treatment. Therefore, the association with the clinical outcome presented in this study served as a hypothesis that might be associated with the possible clinical effects caused by the *CYP2C19* genotype variants. Third, given that there is another method for determining the phenotype, such as measuring the urinary level of 4′-hydroxymephenytoin [16], it would be interesting to conduct a study to deepen our understanding of the association between the *CYP2C19* polymorphism and the phenotype. Fourth, this study only examined the CYP2C19*2 and CYP2C19*3 alleles and did not include CYP2C19*17. CYP2C19*2 and CYP2C19*3 are associated with decreasing or diminished enzyme activity, while CYP2C19*17 variant allele is associated with increased gene expression and increasing enzyme activity and is commonly classified as an ultrarapid metabolizer [49]. CYP2C19*2, CYP2C19*3, and CYP2C19*17 are defined as tier 1 variant alleles due to the well-characterized alteration of CYP2C19 enzyme activity [49]. Further study is needed to examine the mutation in alleles *2, *3, and *17 to provide more accurate data in Indonesia. Currently, the clinical practice of examining *CYP2C19* genotype variants is still unfeasible for use in Indonesia the limited number of facilities capable of performing the test. The results presented in this study might therefore be important for shedding light on the distribution of *CYP2C19* variant alleles at the population level in Indonesia and might be used as a consideration for providing personalized treatment in clinical practice.

## 5. Conclusions

We showed that, in Indonesia, intermediate metabolizers had the highest prevalence, followed by rapid metabolizers and poor metabolizers. Dosage adjustment should be considered when administering PPI-based therapy among ethnicities in Indonesia.

## Figures and Tables

**Table 1:** Demographic Data of Patients and Endoscopic Diagnosis.

Ethnicity	*n*	Age, Years		Sex		Gastritis	Gastric Ulcer	Duodenal Ulcer	GERD *
		Mean ± SD	Range	Male	Female				
Papuan	14	43.4 ± 11.9	21–63	8	6	14 (100)	0 (0.0)	0 (0.0)	0 (0.0)
Batak	27	51.6 ± 16.3	17–78	13	14	24 (88.9)	2 (7.4)	0 (0.0)	1 (3.7)
Balinese	25	46.0 ± 12.6	23–70	15	10	25 (100)	0 (0.0)	0 (0.0)	0 (0.0)
Dayak	10	36.8 ± 11.8	22–54	5	5	9 (90)	0 (0.0)	1 (10.0)	0 (0.0)
Javanese	28	42.5 ± 11.8	19–64	17	11	24 (85.7)	1 (3.6)	0 (0.0)	3 (10.7)
Bugis	37	47.5 ± 14.3	24–76	27	10	30 (81.1)	2 (5.4)	0 (0.0)	5 (13.5)
Chinese	17	46.8 ± 14.1	22–70	10	7	9 (52.9)	0 (0.0)	0 (0.0)	8 (47.1)
Timorese	8	61.0 ± 12.1	44–80	6	2	8 (100)	0 (0.0)	0 (0.0)	0 (0.0)

* GERD, gastroesophageal reflux disease.

**Table 2:** *CYP2C19* Polymorphism Genotyping.

*CYP2* C19*2	*CYP2* C19*3	*CYP2C19* Genotype	Expected Phenotype	*n* (%)	Lower–Upper Proportion (95% CI) **
*1/*1	*1/*1	*1/*1	Rapid Metabolizer	64 (38.5)	31.1–46.4
*1/*2	*1/*1	*1/*2	Intermediate Metabolizer	60 (36.1)	28.8–43.9
*1/*1	*1/*3	*1/*3	Intermediate Metabolizer	9 (5.4)	2.5–10.0
*2/*2	*1/*1	*2/*2	Poor Metabolizer	23 (13.9)	8.9–20.0
*1/*1	*3/*3	*3/*3	Poor Metabolizer	4 (2.4)	0.6–6.0
*2/*2	*1/*3	*2/*3	Poor Metabolizer	1 (0.6)	0.2–3.3
*1/*2	*1/*3	*2/*3	Poor Metabolizer	5 (3.0)	1.0–6.9

** Estimates of the proportion were determined by the Clopper–Pearson Exact method

**Table 3:** *CYP2C19* Genotype among Indonesian Ethnicities.

Ethnicity	CYP2C19*2 (%)			CYP2C19*3 (%)			*CYP2C19* Genotype (%)		
	*1/*1	*1/*2	*2/*2	*1/*1	*1/*2	*2/*2	RM	IM	PM
Papuan	4 (28.6)	5 (35.7)	5 (35.7)	11 (78.6)	1 (7.1)	2 (14.2)	2 (14.3)	4 (28.6)	8 (57.1)
Batak	14 (51.9)	10 (37.0)	3 (11.1)	22 (81.5)	5 (18.5)	0 (0.0)	10 (37.0)	13 (48.1)	4 (14.8)
Balinese	13 (52.0)	8 (32.0)	4 (16.0)	24 (96.0)	1 (4.0)	0 (0.0)	13 (52.0)	8 (32.0)	4 (16.0)
Dayak	5 (50.0)	3 (30.0)	2 (20.0)	9 (90.0)	1 (10.0)	0 (0.0)	4 (40.0)	4 (40.0)	2 (20.0)
Javanese	16 (57.1)	8 (28.6)	4 (14.3)	23 (82.1)	4 (14.3)	1 (3.6)	13 (46.4)	8 (28.6)	7 (25.0)
Bugis	15 (40.5)	19 (51.4)	3 (8.1)	35 (94.6)	2 (5.4)	0 (0.0)	14 (37.8)	19 (51.4)	4 (10.8)
Chinese	7 (41.2)	7 (41.2)	3 (17.6)	15 (88.2)	1 (5.9)	1 (5.9)	5 (29.4)	8 (47.1)	4 (23.5)
Timorese	3 (37.5)	5 (62.5)	0 (0.0)	8 (100)	0 (0.0)	0 (0.0)	3 (37.5)	5 (62.5)	0 (0.0)

RM, rapid metabolizer; IM, intermediate metabolizer; PM, poor metabolizer.

**Table 4:** Association between Sex and Ethnicity and Rapid Metabolizing.

Characteristic	Rapid Metabolizer (%)	OR	95% CI	P-Value
**Sex**				
Male	39 (38.6)	1.006	0.530–1.910	0.984
Female	25 (38.5)	1.000		
**Ethnicity**				
Papuan	2 (14.3)	1.000		
Batak	10 (37.0)	3.529	0.652–19.099	0.143
Balinese	13 (52.0)	6.500	1.199–35.230	0.030 *
Dayak	4 (40.0)	4.000	0.563–28.396	0.166
Javanese	13 (44.4)	5.200	0.978–27.653	0.053
Bugis	14 (37.8)	3.652	0.710–18.785	0.121
Chinese	5 (29.4)	2.500	0.403–15.501	0.325
Timorese	3 (37.5)	3.600	0.454–28.562	0.225

* P < 0.05.

**Table 5:** CYP2C19 and Clinical Outcomes Based on Endoscopy.

CYP2C19	Gastritis		Gastric Ulcer		Duodenal Ulcer	GERD	
	Proportion (%)	95% CI **	Proportion (%)	95% CI **	Proportion (%)	Proportion (%)	95% CI **
RM	55 (38.5)	30.4–47.0	1 (20.0)	0.5–71.6	0 (0.0)	8 (47.1)	23.0–72.2
IM	57 (39.9)	31.7–48.4	4 (80.0)	28.4–99.5	0 (0.0)	8 (47.1)	23.0–72.2
PM	31 (21.7)	15.2–29.3	0 (0.0)	0.0–52.2	1 (100)	1 (5.9)	
Total	143		5		1	17	

RM, rapid metabolizer; IM, intermediate metabolizer; PM, poor metabolizer; GERD, gastroesophageal reflux disease. ** Estimates of the proportion were determined by the Clopper-Pearson Exact method.

## Data Availability

The data that support the findings of this study are available from the corresponding author upon reasonable request.

## Supplementary Materials

The following are available online at https://www.mdpi.com/article/10.3390/biology10040300/s1, Supplementary Table S1: Association between sex and ethnicity to poor metabolizer, Supplementary Table S2: Association between CYP2C19 genotype and histology scores, Supplementary Table S3: Comparison of CYP2C19 allele genotypes among various populations.
