# Supplementary material for: CYP2C19 Polymorphisms in Indonesia: Comparison among Ethnicities and the Association with Clinical Outcomes
Source: Biology (Basel). 2021 Apr 6;10(4):300. doi: 10.3390/biology10040300 (PMC8067412; doi:10.3390/biology10040300)
Supplement: Supplementary file 1 [file biology-10-00300-s001.pdf]

Supplementary Table S1. Association between sex and ethnicity to poor metabolizer

Characteristic	Poor Metabolizer (%)	OR	95% CI	P-Value
**Sex**				
Male	19 (18.8)	1.000		
Female	14 (21.5)	1.185	0.546–2.568	0.668
**Ethnic**				
Bugis	4 (10.8)	1.000		
Papuan	8 (57.1)	11.000	2.498–48.433	0.002 *
Batak	4 (14.8)	1.435	0.325–6.321	0.634
Balinese	4 (16.0)	1.571	0.354–6.972	0.552
Dayak	2 (20.0)	2.062	0.320–13.313	0.447
Javanese	7 (25.0)	2.750	0.717–10.553	0.140
Chinese	4 (23.5)	2.538	0.551–11.691	0.232
Timor	0 (0.0)	–	–	–

* $P < 0.05$

Supplementary Table S2. Association between *CYP2C19* genotype and histology scores

Histology	Total	*n* RM	*n* IM + PM	P-Value
Neutrophil infiltration	43	17	26	0.999
Monocyte infiltration	69	23	46	0.262
Antrum predominant gastritis	49	20	29	0.729
Corpus predominant gastritis	10	3	7	0.742

RM, rapid metabolizer; IM, intermediate metabolizer; PM, poor metabolizer.

Supplementary Table S3. Comparison of *CYP2C19* allele genotypes among various populations

Population	*CYP2C19* Genotype (%)			Reference	Year
	RM	IM	PM		
Indonesian	38.5	41.6	19.9	This study	2021
Japanese	34.9	46.3	18.8	Kubota et al [16]	1996
Japanese	33.7	48.7	17.6	Miyoshi et al [17]	2001
Chinese	34.8	50.4	14.8	Lin et al [14]	2017
Korean	28.6	52.9	18.5	Oh et al [36]	2008
Korean	41.3	46.8	11.9	Kang et al [49]	2008
Thai	40.6	49.0	10.4	Jainan et al [19]	2014
Turkey	71.7	23.6	4.7	Ozdil et al [23]	2009
Turkey	78.0	19.5	2.5	Ormeci et al [15]	2016
Egyptian	65.0	26.0	9.0	Settin et al [24]	2013
Poland	80.0	20.0	0.0	Gawronska-Szklarz et al [50]	2005
Italian	81.1	17.5	1.4	Sapone et al [51]	2003

RM, rapid metabolizer; IM, intermediate metabolizer; PM, poor metabolizer.
